# Function of the Nuclear Transport Machinery in Maintaining the Distinctive Compositions of the Nucleus and Cytoplasm

**DOI:** 10.3390/ijms23052578

**Published:** 2022-02-25

**Authors:** Murray Stewart

**Affiliations:** MRC Laboratory of Molecular Biology, Francis Crick Avenue, Cambridge Biomedical Campus, Cambridge CB2 0QH, UK; ms@mrc-lmb.cam.ac.uk; Tel.: +44-(0)1223-267074

**Keywords:** nuclear pore, nuclear transport, nuclear transport, nucleoporin, thermal ratchet

## Abstract

Although the separation of transcription and translation, mediated by the nuclear envelope, is the defining characteristic of Eukaryotes, the barrier between the nuclear and cytoplasmic compartments needs to be semipermeable to enable material to be moved between them. Moreover, each compartment needs to have a distinctive complement of macromolecules to mediate specific functions and so movement between them needs to be controlled. This is achieved through the selective active transport of macromolecules through the nuclear pores that stud the nuclear envelope, and which serve as a conduit between these compartments. Nuclear pores are huge cylindrical macromolecular assemblies and are constructed from the order of 30 different proteins called nucleoporins. Nuclear pores have a central transport channel that is filled with a dense network of natively unfolded portions of many different nuclear pore proteins (nucleoporins or nups). This network generates a barrier that impedes, but does not entirely prevent, the diffusion of many macromolecules through the pores. The rapid movement of a range of proteins and RNAs through the pores is mediated by a range of transport factors that bind their cargo in one compartment and release it in the other. However, although as their size increases the diffusion of macromolecules through nuclear pores is progressively impaired, additional mechanisms, including the binding of some macromolecules to immobile components of each compartment and also the active removal of macromolecules from the inappropriate compartment, are needed to fully maintain the distinctive compositions of each compartment.

## 1. Introduction 

The defining characteristic of Eukaryotic cells is the separation of translation from transcription that is mediated by the double membrane of the nuclear envelope. This separation enables transcripts to be modified by capping, splicing and polyadenylation in the nucleus before they are translated into proteins on ribosomes in the cytoplasm. Although the movement of macromolecules such as proteins and RNAs between the nuclear and cytoplasmic compartments is facilitated by nuclear pores, a level of selectivity is necessary to maintain the different populations of macromolecules present in each compartment. The nuclear transport system together with the diffusion barrier present in the nuclear pore transport channel contribute to the generation of this selectivity.

## 2. Nuclear Transport Machinery

The nuclear transport machinery is based on the nuclear pores that stud the nuclear envelope together with a series of soluble transport factors and associated proteins that mediate the selective active transport of proteins and nucleic acids between the nuclear and cytoplasmic compartments. The basic features of these two components will first be reviewed before discussing how they combine to generate the distinctive compositions of the nuclear and cytoplasmic compartments.

### 2.1. Nuclear Pores

**Figure 1:**
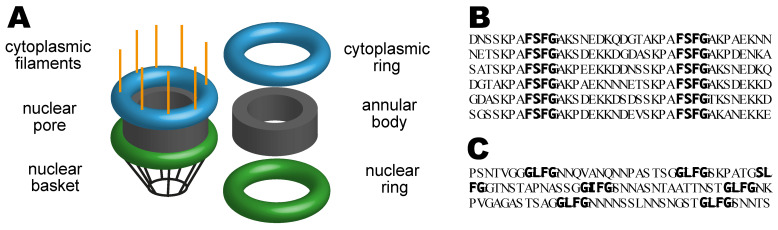
(**A**) Highly schematic illustration of nuclear pore structure. Nuclear pores span the double membrane of the envelope and are based on a central annular core (sometimes referred to as a central ring [10] and which is constructed from two similar halves) that is sandwiched between inner (nucleoplasmic) and outer (cytoplasmic) rings. Appendages also extend into the cytoplasm and the nucleus (for clarity these have been omitted from the exploded view). Complementary X-ray crystallography and cryo-EM studies have established the arrangement of the nuclear pore proteins (nucleoporins or “nups”) in *S. cerevisiae* and other species [1,2,3,4,5,6,7,8,9,10]. Nuclear pores serve as a conduit between the nuclear and cytoplasmic compartments, with macromolecules such as proteins and RNAs moving through the central channel. Many nucleoporins (“FG-nucleoporins”) contain long regions containing characteristic sequence repeats based on cores rich in Phe (F) and Gly (G) that lack secondary structure (or are “natively unfolded”) and which protrude into the central channel where they form a dense mesh that impairs the movement of macromolecules. The fibrous nuclear basket and cytoplasmic filaments have been omitted for clarity in the exploded image. (**B**) Portion of the *S. cerevisiae* FG-nucleoporin Nup1 showing FG rich cores (bold), here containing FSFG (bold), interspersed with linkers of variable sequences that generally lack hydrophobic residues. (**C**) Portion of the sequence of *S. cerevisiae* Nup116 that contains cores based on a GLFG motif (bold). Here the lengths of the regions between the FG cores are more variable than in Nsp1.

Nuclear pores (sometimes also called “nuclear pore complexes” or “NPCs” for short) are based on an annular scaffold that has 8-fold rotational symmetry with a central aqueous transport channel that connects the nuclear and cytoplasmic compartments. The structure of the annular scaffold is conveniently thought of as a central annular core sandwiched between cytoplasmic (“outer”) and nucleoplasmic (“inner”) rings (Figure 1A) and complementary studies using cryo-EM and X-ray crystallography have developed detailed models of the way in which the chains of the various nuclear pore proteins (nucleoporins or “nups”) are arranged in these components and how the pores are attached to the nuclear envelope membrane [1,2,3,4,5,6,7,8,9,10]. Fibrous appendages project from both the cytoplasmic and nucleoplasmic faces of the nuclear pores (Figure 1A), with those in the nucleus contributing to the formation of a distinctive nuclear basket [11]. These fibrous appendages function as binding sites for a range of factors involved in both transport and gene expression [12,13]. The annular bodies of nuclear pores appear to have a degree of flexibility and, when imaged by cryo-EM in whole cells, appear to reversibly constrict on cellular energy depletion or osmotic stress, whereas they are dilated in exponentially growing cells [14], paralleling earlier observations that indicated a variation in limiting diameter in different cellular states [15] and the considerable variation in nuclear pore diameter seen in intact cells using cryo-EM of milled sections [16].

Nuclear pores are constructed from multiple copies of ~30 different nucleoporin proteins. A number of these nucleoporins contain regions that possess long intrinsically disordered (or natively unfolded) domains that protrude into the central channel where they generate a dense meshwork that impedes the diffusion of macromolecules between the nuclear and cytoplasmic compartments [17,18,19]. These intrinsically disordered chains contain characteristic sequence repeats based on small (2–5 residue) cores rich in phenylalanine (F) and glycine (G) residues separated by short linkers that are generally more hydrophilic and are referred to as “FG-nucleoporins”. Figure 1B,C illustrate regions in the *S. cerevisiae* FG-nucleoporins Nsp1 and Nup116 that show these sorts of patterns. Characteristic sequence motifs in the core of the FG-nucleoporin repeats include FxFG (where x is a small residue such as serine), GLFG, and FG [18,19,20,21].

The densely packed natively unfolded chains of the FG-nucleoporins generate a barrier to the diffusion of macromolecules between the nuclear and cytoplasmic compartments and also function as transient binding sites for the nuclear transport factors (or “carriers”) that mediate the active import and/or export of a range of macromolecules [12,17,19,22,23,24,25,26,27]. Interactions between the transport factors and the FG-nucleoporins enable them to enter the nuclear pore transport channel and carry protein and RNA cargoes between the nuclear and cytoplasmic compartments (reviewed by [12,24]). Although natively unfolded proteins occupy a larger volume than folded proteins [28], it is not clear the extent to which the crowding within the nuclear pore transport channel may be greater than that present in the bulk of the nucleus or cytoplasm. Within intact cells, the highly crowded environment of cytoplasm tends to restrict the FG-nucleoporin chains to the nuclear transport channel. However, the FG-nucleoporins certainly appear to protrude out into the cytoplasm when most of the cytoplasm has been removed in permeabilized cells [17] and it is not clear whether this might influence nuclear transport when permeabilized cells are used in model studies. The molecular crowding in the nuclear pore transport channel is increased by the considerable number of transport factors that are also present in the nuclear pore transport channel [29,30]. 

### 2.2. The Nuclear Pore Diffusion Barrier 

Although small molecules are able to diffuse freely through the nuclear pore channel, the passage of macromolecules that do not interact with the FG-nucleoporins is impaired progressively as their size increases [31,32,33] until particles are larger than ~120 Å diameter can no longer penetrate. It has been difficult to define the precise manner in which the FG-nucleoporins generate this diffusion barrier, although it appears likely that several different mechanisms, including cohesive interactions between FG-nucleoporins and entropic restrictions similar to those involved in molecular brushes, contribute to this function. Although the cytoplasm and nucleoplasm are also crowded environments, the entropic effects would be expected to be greater for the natively unfolded nature of the FG-nucleoporin chains relative to the generally folded proteins present in the cytoplasm or the contents of the nucleoplasm.

In the ‘‘selective phase’’ model [19,34,35] it is proposed that cohesive interactions between the FG-nucleoporins generate a meshwork in the pore transport channel that acts as a molecular sieve that impairs the diffusion of large molecules. Conversely, the transport carrier proteins are able to penetrate the meshwork because the energy liberated from their binding to hydrophobic cores of the FG-nucleoporins is sufficient to overcome the breaking of cohesive contacts among the FG-nucleoporins [19,35] needed to facilitate their movement through the transport channel. Cohesive interactions among the FG-nucleoporins are thought to derive from a combination of hydrophobic, electrostatic, and other attractions, and appear to contribute to the generation of a dense meshwork within the central nuclear pore channel that impairs the movement of macromolecules [18,19,22,23,34,36,37,38]. 

Alternatively, the ‘‘brush’’ model or the ‘‘virtual gate’’ model, proposes that the diffusion barrier results from the entropic cost of macromolecules entering into the densely-packed network of natively disordered nucleoporin chains in the pore transport channel. The dense packing of the natively unfolded FG-nucleoporin chains in the pore transport channel will tend to limit their conformational entropy in ways analogous to that seen in an Alexander-deGennes polymer brush. The entropy of these chains would be expected to be further reduced when the packing density was increased by the presence of additional macromolecules in the channel and this effect has been proposed to also contribute to the nuclear pore diffusion barrier [39].

Although the assembly of intrinsically disordered proteins chains that line the central channel of nuclear pores is central to understanding the generation of the specific compositions of nucleus and cytoplasm, it remains technically difficult to establish directly in intact pores the morphology of this assembly and precisely how it mediates both the pore barrier function and also the selectivity of nuclear transport. Although a detailed picture of the arrangement of the protein chains in the annular structural scaffold of nuclear pores has been generated using cryo-EM, crystallography, and computational methods [1,2,3,4,5,6,7,8,9], it has not been possible to determine the way in which the intrinsically disordered chains of the FG-nucleoporins that pack the transport channel are arranged. Consequently, it has been necessary to employ in vitro experimental systems together with theoretical and computational modeling to obtain insights. To this end, several in vitro systems, based primarily on the production of gels or of dense arrays on a surface, have been employed to investigate the contributions made by cohesion, molecular crowding, and molecular brush mechanisms to the generation of the barrier function of nuclear pores. 

Extensive bioinformatic analyses, based on the spatial clustering of features such as FG repeat cores and charges within the intrinsically disordered regions of FG-nucleoporin chains indicated a group of conserved spatial features, including the separation, localization, and ordering of FG cores and charged residues [20,21,40,41]. Coarse-grained simulations of both individual nucleoporins and grafted rings of nucleoporins mimicking the in vivo geometry of nuclear pores, supplemented with polymer brush modeling, indicated that different regions or blocks of an individual FG-nucleoporin protein chain can have different forms of disorder [40,41]. Moreover, at the individual protein level, this block structure appeared to be critical to generating a polymer brush architecture that can exhibit different morphologies depending on the interaction energy between the FG repeat cores of different nucleoporins. It was proposed that, because the interactions between FG repeat cores may be modulated by interactions with transport carrier proteins, transitions between brush morphologies could contribute to facilitating transport through the pores [40,41]. Consistent with this proposal, work that examined the rotational mobility of fluorescent probes at various locations within the FG-network indicated that the internal organization of the FG-nucleoporins within the nuclear pore transport channel generated multiple dynamic environments that had different local properties [42]. 

In vitro experiments have shown that sufficiently strong cohesiveness may drive the formation of dense, gel-like FG-nucleoporin aggregates through a mechanism similar to liquid-liquid phase separation [18,19,22]. Transport carrier proteins loaded with fluorescent cargoes are able to diffuse into these gels, whereas other proteins are excluded. Assemblies of FG-nucleoporins grafted onto planar surfaces or in nuclear pore-like nanopores have also been used to investigate the arrangement of these intrinsically disordered chains and how they are impacted by interactions with transport carrier proteins, as well as how these interactions may influence their permeability and transport properties [43,44,45,46,47]. These polymer brush like assemblies of FG-nucleoporins also allow transport carrier proteins loaded with cargoes to penetrate into their interior while excluding other proteins [23,46,48,49,50].

Coarse-grained polymer theory and numerical simulations [51] have indicated that, in high-density FG-nucleoporin assemblies, an increase in cohesiveness decreases their permeability, whereas in low-density assemblies, permeability increases with cohesiveness. Overall, these effects can be explained by considering the free-energy cost associated with penetrating the FG-nucleoporin assemblies. These results support a model in which assemblies of low-density, low-cohesiveness FG-nucleoporins at the periphery of the pores enhance the targeted capture of the transport proteins and expedite their partitioning into the interior of the pore, whereas the selectivity barrier is generated by the high-density assembly of the centrally located, highly cohesive FG-nucleoporins [51].

These studies have been extended by developing computational and analytical models that include contributions from the heterogeneities in FG-nucleoporin sequences, that are considered as essentially a string of cohesive and noncohesive polymer units, together with contributions from the surface of the transport carrier proteins that interact with the FG cores [52]. Comparing these models with experimental data on single-molecule interactions between FG-nucleoporins and transport carrier proteins indicates that, although the heterogeneous nature of FG-nucleoporins and carriers does contribute to the strength of equilibrium binding, these features make a much larger contribution to the kinetics of binding and unbinding. These models indicate how binding equilibria and kinetics depend on the distribution of both cohesive blocks in the FG-nucleoporin sequences and of the binding pockets on the carrier proteins and that multivalency is important. This work also suggests that single-molecule binding kinetics has a relatively small influence on the diffusion of transport carrier proteins in polymer melts consisting of FG-nucleoporin-like sequences [52]. 

### 2.3. Active Transport through Nuclear Pores

For many macromolecules, the rate of diffusion between the nuclear and cytoplasmic compartments is too slow and so the rate at which they transit through nuclear pores is increased by a range of nuclear transport factors or “carriers” that bind their cargoes in the donor compartment, diffuse rapidly through the pores, and then release the cargo in the target compartment [12,24,53]. Proteins that are actively transported through nuclear pores are generally recognized by their carriers through small sequence motifs, such as the classical nuclear localization signal (NLS) recognized by importin-α [54], or the nuclear export signal (NES) recognized by CRM1 [53]. 

It is generally considered that the diffusion of macromolecules greater than about 40 kD through nuclear pores is impaired and so the rate of diffusion of many macromolecules through nuclear pores is too slow and needs to be enhanced by specific transport systems in which protein transport factors or “carriers” function to mediate their exchange between the nuclear and cytoplasmic compartments. A number of different nuclear transport systems function to overcome the barrier function of the FG-nucleoporins and greatly increase the rate of both the import and export of a broad range of proteins and the export of fully-processed RNAs. The import and export of proteins into the nucleus is mediated primarily by transport factors of the importin-β (β-karyopherin) superfamily, whereas mRNA export is mediated mainly by the NXF1:NXT1 family [12,24,53,55]. The energy required to power nucleocytoplasmic transport is supplied indirectly and is used to assemble and disassemble the cargo:carrier complexes rather than actually moving these complexes through the nuclear pore transport channel itself [24]. Thus, although nuclear transport is an example of active transport, energy is not used for the movement through the pores but is instead used to mediate the binding and release of cargoes and so is an example of a thermal ratchet [56,57], whereby the energy is used rectify thermal motion. For β-karyopherin-based transport pathways, the energy is supplied by RanGTP that orchestrates the binding and release of cargoes in the appropriate compartment. 

**Figure 2:**
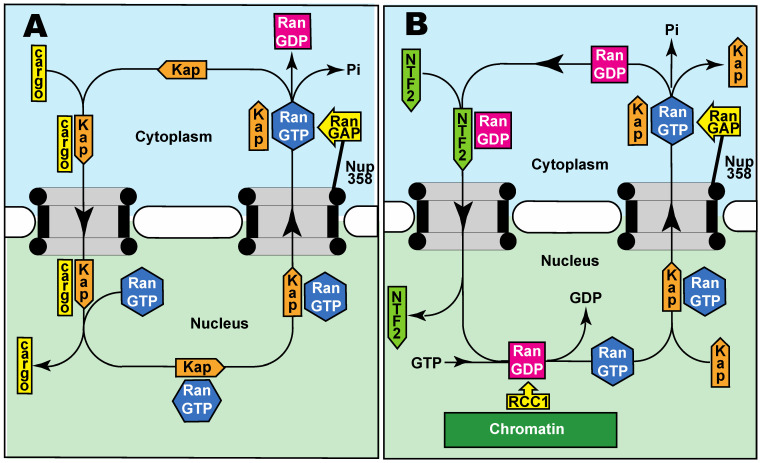
Highly schematic illustration of nuclear transport mechanisms. (**A**) Nuclear protein import whereby cargo proteins are bound by a karyopherin carrier (Kap) of the importin-β superfamily in the cytoplasm (often using an adapter protein such as importin-α or by “piggy-backing” on another protein that is being imported) that is able to circumvent the barrier generated by FG-nucleoporins (grey) in the nuclear pore transport channel and facilitate movement into the nucleus, where RanGTP binding releases the cargo. The karyopherin:RanGTP complex is then recycled to the cytoplasm where the GTPase activating protein RanGAP activates the RanGTPase, releasing RanGDP and freeing the karyopherin for a further import cycle. Energy, therefore, is used to rectify thermal motion rather than move material through the pores. (**B**) NTF2 then binds RanGDP and recycles it to the nucleus where the guanine nucleotide exchange factor RCC1 recharges it with GTP. To maintain the RanGTP gradient, RanGAP is retained in the cytoplasm by binding to Nup358, whereas RCC1 is retained in the nucleus bound to chromatin.

For nuclear import (Figure 2A), β-karyopherins bind their cargoes in the cytoplasm (frequently using an adapter protein such as importin-α in the case of importin-β) and, after diffusing through nuclear pores [12,24,58], this complex is dissociated by RanGTP binding to the β-karyopherin, a process that may be accelerated by specific nucleoporins such as Nup1 and Nup2 located at the cytoplasmic face of the nuclear pore [13,59]. The β-karyopherin:RanGTP complex then returns to the cytoplasm where the GTPase activity of Ran is stimulated by its activating protein (RanGAP) generating RanGDP that then dissociates from the β-karyopherin, freeing it for another round of nuclear import. The RanGDP so generated is then recycled to the nucleus by NTF2, where the Ran guanine nucleotide exchange protein, RCC1, recharges it with GTP (Figure 2B). Conversely, the principal nuclear protein export carrier CRM1 complexed with RanGTP binds its cargo in the nucleus and subsequently releases the cargo in the cytoplasm following GTP hydrolysis stimulated by RanGAP [53]. Cargoes are generally recognized by the appropriate carrier through specific sequence motifs, such as the classic nuclear protein import nuclear localization sequences [12,24,54,60]. Also, increasing the number of these signal sequences increases the rate of transport of larger cargoes [61,62]. The nuclear export of most mRNAs instead employs the NXF1:NXT1 heterodimer as a carrier and here the binding and unbinding of cargo are orchestrated by altering the RNA structure using ATP-powered DEAD-box helicases in the nucleus and cytoplasm [63,64]. In addition to nuclear pores facilitating their export to the cytoplasm, the nuclear basket of the pores functions as a scaffold for several components of the mRNA maturation machinery, most notably in yeast [55,63,64,65].

Although most transport systems are generally described as having the transport factors, such as importin-β or CRM1, released into the cytoplasm or nucleoplasm at the end of a cycle, it is probably more likely that they cycle within the pores themselves because of their affinity for the FG-nucleoporins. Indeed, in living cells, the nuclear pores appear to contain a considerable number of transport factors [29] that may be cycling between the nuclear and cytoplasmic compartments, binding and releasing their cargos at the respective faces of the pores while not often moving into the nuclear or cytoplasmic compartments. Moreover, cargo:carrier complexes are sometimes dissociated during their actual passage through the pores or may also return to their original compartment so that the efficiency of the transport system is somewhat lower than is often thought [66,67].

The carrier proteins are able to overcome the diffusion barrier generated by the FG-nucleoporins in the nuclear pore transport channel through binding to their phenylalanine-rich cores and this is able to reduce the FG-nucleoporin barrier function sufficiently to enable the carriers (bond to cargo or RanGTP or free) to move rapidly back and forth between the nuclear and cytoplasmic compartments. The precise mechanism by which the carriers overcome the FG-nucleoporin barrier remains controversial and probably involves contributions from the interaction energy with the FG cores reducing the cohesion between cores facilitating their forming a less cohesive meshwork.

## 3. Distinctive Nuclear and Cytoplasmic Compositions

A crucial function of the nuclear transport machinery is to generate and maintain the distinctive compositions of the nucleus and cytoplasm. In this context, the primary requirements of the nuclear transport system are to exclude ribosomes from the nucleus, retain chromosomes in the nucleus, and retain transcripts in the nucleus until they have been fully processed. However, many other cellular functions require the presence of key macromolecules in either the nucleus or the cytoplasm, and many signaling pathways, for example, require the movement of key regulators between the compartments at appropriate times. 

The overall size of ribosomes and chromosomes appears to be sufficient to prevent their movement through nuclear pores, whereas the conformational flexibility of transcripts appears to enable these often quite large assemblies to passage through the pores once they have been released by the nuclear modification machinery. An extreme example is the export of Balbiani rings [68]. Remarkably, apparently intact, cone-shaped HIV-1 capsids have been shown to pass through nuclear pores in T-cells before they are disassembled in the nucleus [69]. However, although the rate of movement through nuclear pores of macromolecules decreases as their size increases [31,32], particles smaller than ~120 Å diameter can still diffuse between the cytoplasmic and nuclear compartments [31] and so additional mechanisms are required, that can involve the export of the inappropriately located macromolecule from the compartment or tethering the molecules to a large component to prevent their moving. 

The extent to which the diffusion barrier generated by the FG-nucleoporins determines the different compositions of the nuclear and cytoplasmic compartments is not completely clear. Although the rate of diffusion of macromolecules through nuclear pores decreases as their size increases [31,32], this alone is insufficient to maintain the different compositions of the nuclear and cytoplasmic compartments because it appears that particles smaller than ~120 Å can still diffuse through the pores on a time scale of at least minutes [31] and so these macromolecules would eventually equilibrate between the two compartments. Therefore, in addition to accelerating movement of required macromolecules, the transport machinery also functions to remove molecules that have diffused into the inappropriate compartment rather than the FG-nucleoporin barrier actually preventing their entry. 

Although the degree to which movement through nuclear pores is impaired increases with the size of the macromolecule [31,32,33], a diffusion limit of ~40 kD is often described for proteins. However, much larger particles are still able to diffuse through the pores, whereas some smaller proteins need their passage to be accelerated. For example, work with BSA-coated colloidal gold particles [31] indicated that particles having diameters up to ~120 Å were able to diffuse through nuclear pores, whereas conversely smaller molecules, such as Ran/Gsp1 (Mr 25,000 kDa), a key component of β-karyopherin-based transport, require transport factors (NTF2 in the case of RanGDP—see Figure 2B) to increase their rate of movement between the cytoplasm and nucleus [70,71]. In addition to the size of macromolecules, their surface properties also influence diffusion through nuclear pores, with surface negative charges and lysine residues slowing diffusion whereas hydrophobic surface residues increase the rate of diffusion [18]. The maximum size of particles that can be actively transported through nuclear pores is substantially greater than the diffusion limit. For example, gold particles coated with nucleoplasmin up to a limiting diameter of over 300 Å can be imported, as can large virus capsids [15,69,72]. 

Although the nuclear pore barrier alone is unable to prevent inappropriate localization of key macromolecules in either the nuclear or cytoplasmic compartments, this can be achieved by supplementing the barrier function by either attaching components to an immobile feature, such as the cytoskeleton, chromatin or organelles, or by removing inappropriately localized components using active transport. 

The two key components that orchestrate the nucleotide state of Ran, for example, need to be located exclusively in the appropriate compartment to maintain the RanGTP gradient that is essential for β-karyopherin-based nuclear transport (Figure 2), and indeed the direction of transport is reversed if their locations are interchanged [73]. This localization is achieved by the guanine nucleotide exchange factor, RCC1/prp20, being bound to chromatin in the nucleus [74], whereas the GTPase activating protein, RanGAP, is attached to NUP358 that forms the filaments that protrude from the cytoplasmic face of nuclear pores [75].

**Figure 3:**
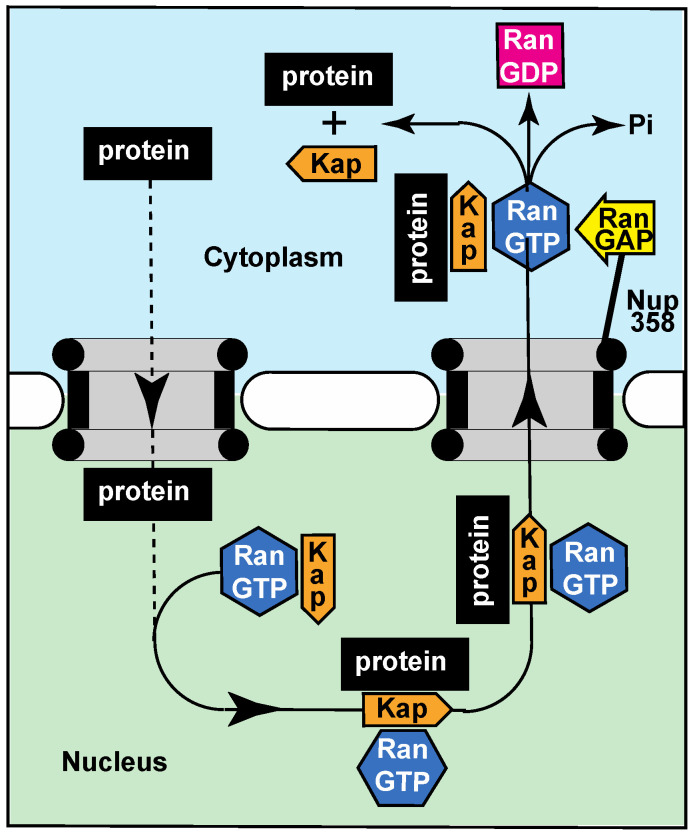
As the barrier generated by the FG nucleoporins in the nuclear pore transport channel only impairs (rather than prevents) the diffusion of macromolecules smaller than ~120 Å diameter into the nucleus, those that enter the nucleus inappropriately (dashed line) are rapidly returned to the cytoplasm by exportin karyopherins (Kap) bound to RanGTP (full line). When the protein:carrier:RanGTP complex reaches the cytoplasm, RanGAP stimulates the RanGTPase, releasing both the exportin and the cargo.

As the nuclear pore diffusion barrier cannot completely inhibit many proteins (including those larger than ~40 kD) diffusing inappropriately into the nucleus, an alternative strategy is to continuously remove these molecules by exporting them back to the cytoplasm (Figure 3). For example, because its cytoplasmic concentration is so high and it is relatively small, G-actin (often complexed with other proteins such as cofilin) can diffuse through nuclear pores but, although G-actin functions as a component of the DNA repair machinery [76], the concentration of free G-actin in the nucleus is maintained at a low level by its being efficiently exported back to the cytoplasm [77,78]. Similarly, nuclear proteins that diffuse back to the cytoplasm are rapidly returned to the nucleus by the import machinery (Figure 2A).

Components of many signaling pathways, such as the androgen receptor, glucocorticoid receptor, and NF-κB, and also components of the MAP kinase pathway, shuttle between the nucleus (where they activate expression of target genes) and the cytoplasm, where they are retained in the absence of a signal or where they detect the appropriate signal (reviewed by [79,80]). For example, the NF-κB (nuclear factor κB) family transcription factors, which are master regulators of immune and inflammatory processes in response to both injury and infection, are sequestered in the cytosol by their inhibitor IκB (inhibitor of NF-κB) proteins. Following stimulation of innate immune receptors and cytokine receptors, a series of events lead to the phosphorylation of IκBs resulting in their proteasomal degradation and the release of NF-κB for nuclear translocation and activation of gene transcription [79]. Inappropriate binding to components of the donor compartment can also be impaired by the carriers to which they are bound also acting as a chaperonin as seen, for example, with histones [81]. Although signaling molecules such as these are larger than 40 kD, they can still occasionally diffuse through nuclear pores (see Section 2.2 above), but any that enter the inappropriate compartment would be returned rapidly by the nuclear transport machinery.

Overall, the nuclear pore diffusion barrier is effective at separating chromosomes and transcription from ribosomes and translation that is necessary for Eukaryotic cell function by preventing the movement of ribosomes and chromosomes through nuclear pores. Although some smaller macromolecules may be able to diffuse into an inappropriate compartment, they can be easily returned by the active transport machinery. This is not to say that the diffusion barrier is ineffective because, although it may not inhibit completely the diffusion of some molecules into an inappropriate compartment, it does slow their diffusion sufficiently so that a much smaller amount of energy is required to maintain the appropriate distribution and indeed many disease states, including cancer and neurodegeneration, arise when either the nuclear pore diffusion barrier or nucleocytoplasmic transport is altered [82,83,84,85,86].
